# Dl-3n-butylphthalide reduces epileptiform activity through GluA2-lacking calcium-permeable AMPARs in epilepsy models

**DOI:** 10.18632/oncotarget.21529

**Published:** 2017-10-05

**Authors:** Qin Yang, Yi-Da Hu, Xue-Feng Wang, Fang-Shuo Zheng

**Affiliations:** ^1^ Department of Neurology, The First Affiliated Hospital of Chongqing Medical University, Chongqing Key Laboratory of Neurology, Chongqing, 400016, China; ^2^ Center of Epilepsy, Beijing Institute for Brain Disorders, Beijing, 100101, China

**Keywords:** dl-3n-butylphthalide, epileptiform activity, CP-AMPARs, patch-clamp technique, epilepsy models

## Abstract

Epilepsy is the most prevalent chronic neurological disorder, and its pathological mechanism indicates that an imbalance between excitatory and inhibitory neurotransmission leads to neuronal hyperexcitability. Previous studies have suggested that dl-3n-butylphthalide (NBP) regulates the excitatory neurotransmitter glutamate in the brains of epileptic mice, however, the mechanisms are unknown. We investigated behavioral and electrophysiological factors in rats using NBP. In an *in vivo* pentylenetetrazole (PTZ)-induced epileptic seizure animal model, NBP decreased the generalized tonic-clonic seizure (GTCS) severity. In an acute hippocampal slice 4-aminopyridine (4-AP) epilepsy model *in vitro*, NBP decreased the epileptiform activity and miniature excitatory postsynaptic current (mEPSC) amplitude; there was no change in the miniature inhibitory postsynaptic current (mIPSC) amplitude or frequency. This effect suggested changes in excitatory synaptic transmission, which was altered through postsynaptic GluA2-lacking calcium-permeable AMPA receptors (CP-AMPARs). These findings showed that NBP suppressed epileptiform activity in these epilepsy models and provided the first detailed electrophysiological analysis of the impact of NBP in epilepsy models, which may be employed in future experimental or clinical therapies for patients with epilepsy.

## INTRODUCTION

The carbonyl compound 3n-butylphthalide (butylphthalide) is one of the chemical constituents of celery essential oil, along with sedanolide, which is obtained from celery leaves and stalks and is primarily responsible for the aroma and taste of celery [1]. Previous studies have suggested that 3*n*-butylphthalide may be useful for the effective treatment of hypertension [2, 3] and cerebral ischemia, the alleviation of oxidative stress caused by chronic cerebral ischemia, the improvement of cholinergic function, and the inhibition of amyloid beta accumulation, thereby improving cerebral neuronal injury and cognitive deficits and significantly inhibiting platelet aggregation [4–6]. This compound also possesses anti-thrombotic and anti-inflammatory effects and reduces neural apoptosis [7, 8]. Furthermore, 3*n*-butylphthalide may ameliorate microcirculation disorders and reduce the blood-brain barrier damage and cerebral edema caused by diffuse brain injury [4, 9]. Dl-3*n*-butylphthalide has been approved by the State Food and Drug Administration of China for the treatment of cerebral ischemia since 2002 [10, 11].

Epilepsy is the most prevalent chronic neurological disorder and is characterized by the occurrence of chronic spontaneous recurrent seizures [12]. The pathological mechanism of epilepsy indicates an imbalance between excitatory and inhibitory neurotransmission, which leads to neuronal hyperexcitability [13]. This condition is refractory to current therapies in approximately 30% of patients [14]. Most current antiepileptic drugs modulate synaptic transmission to reduce neuronal excitability by targeting voltage-gated ion channels. However, the therapeutic benefit of these treatments is limited by a lack of efficacy in some refractory patients and their side effect profiles [15]. Studies in acute epileptic mouse models generated through the intraperitoneal injection of 3n-butylphthalide suggest that this compound regulates the balance of excitatory and inhibitory systems by reducing the glutamate and glutamate /GABA contents in the brains of epileptic mice, which may relieve seizures [16, 17]. However, the detailed regulatory mechanisms responsible for this action of 3n-butylphthalide remain unknown. Using a pentylenetetrazole (PTZ)-induced epileptic seizure animal model *in vivo* and electrophysiological techniques in *in vitro* 4-aminopyridine (4-AP; 100 μM) acute hippocampal slice epilepsy models, we revealed that NBP decreased neuronal hyperexcitability through postsynaptic GluA2-lacking calcium-permeable AMPA receptors (CP-AMPARs).

## RESULTS

### NBP reduces PTZ-induced epileptic seizures

**Figure 1:**
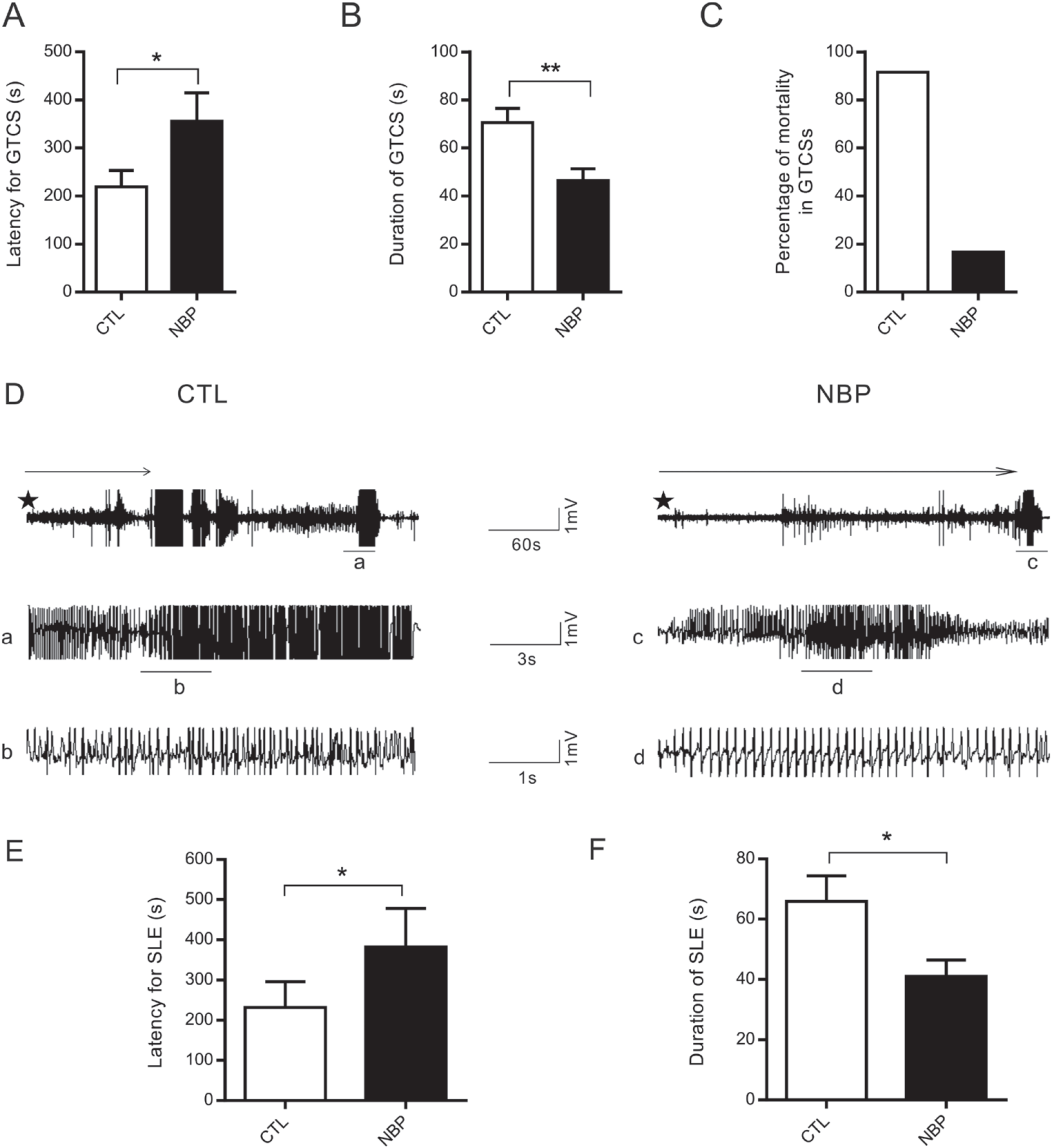
The effect of acute NBP administration (100 mg/kg, i.p.) on the seizures and LFP recordings of rats induced by PTZ (70 mg/kg, i.p.). (**A**) Latency to generalized tonic-clonic seizures (GTCSs) (CTL, *n* = 11; NBP, *n* = 9). (**B**) Duration of GTCSs (CTL, *n* = 11; NBP, *n* = 9). (**C**) Percentage of mortality rate in PTZ-induced seizures assessed for 60 min (CTL, *n* = 12; NBP, *n* = 12). (**D**) Representative traces of seizure-like events (SLEs) with LFP recordings in rats; (A) (B) (C) and (D) respectively represent the expansions of the tracings of SLEs. The star represents the start of LFP recording, and the arrow represents latency of seizure-like events with GTCSs onset. (**E**-**F**) Summary graph of latency (F) and duration (**G**) of SLEs (CTL, *n* = 7; NBP, *n* = 7). The data are presented as the means±SEM. ^*^ *P* < 0.05, ^**^ *P* < 0.01 compared with the NBP-treated group (paired Student's *t*-test).

PTZ is a convulsant chemical agent that has been frequently used in experimental animal models of seizure induction [18]. To determine whether NBP affects seizures, we performed PTZ-induced acute epileptic seizure model *in vivo*. After rats were pretreated with vehicle or 100 mg/kg NBP for 2 h, they were treated with 70 mg/kg PTZ. Treatment with NBP increased the latency to generalized tonic-clonic seizures (GTCSs) (CTL: 218.90 ± 33.75 s, *n* = 11; NBP: 355.70 ± 59.09 s, *n* = 9; *P* = 0.049, Student's *t*-test; Figure 1A), reduced the duration of GTCSs (CTL: 70.62 ± 5.90 s, *n* = 11; NBP: 46.44 ± 4.87 s, *n* = 9; *P* = 0.0066, Student's *t*-test; Figure 1B), and prevented seizure-related mortality (CTL, *n* = 12; NBP, *n* = 12; Figure 1C). To measure the local field potentials (LFPs) between the PTZ-treated rats and PTZ+NBP-treated rats, we recorded hippocampal LFPs in the CA1 region (Figure 1D). Seven days after recovery from surgery, the rats that received PTZ and the rats that received PTZ+NBP experienced a GTCS episode (with behaviors corresponding to stage 5) within the first 60 min after PTZ administration, with epileptiform activity simultaneously evolving in all channels out of background. The results showed that NBP prolonged the latency to seizure-like events (CTL: 160.3 ± 31.50 s, *n* = 7; NBP: 382.1 ± 96.12 s, *n* = 7; *P* = 0.0412, Student's *t*-test; Figure 1E) and shortened the duration of seizure-like events (CTL: 65.88 ± 8.52 s, *n* = 7; NBP: 41.10 ± 5.32 s, *n* = 7; *P* = 0.0297, Student's *t*-test; Figure 1F). Therefore, the results indicated that NBP retarded seizure precipitation and reduced the severity of seizures and seizure-like events in an *in vivo* PTZ-induced epileptic seizure model.

### NBP reduces the excitability of pyramidal neurons under epileptic conditions

**Figure 2:**
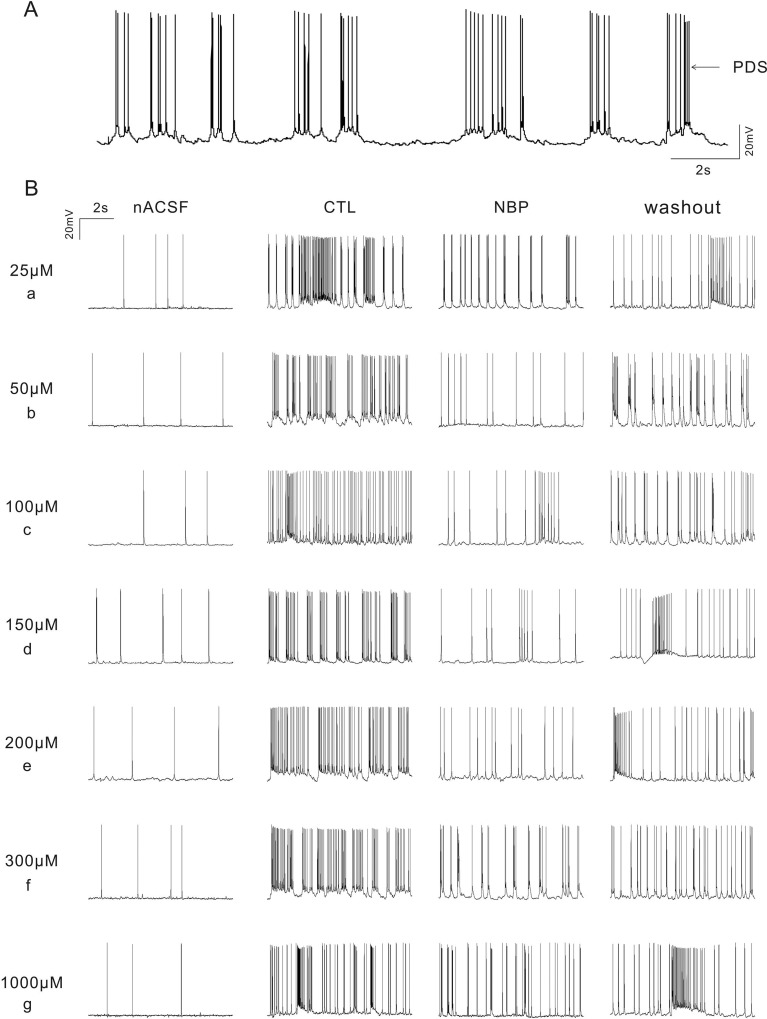
Spontaneous firing frequency and typical PDS of CA1 pyramidal neurons in a 4-AP epilepsy model (**A**) Representative typical traces of PDS. (**B**) Representative traces of spontaneous firing frequency and PDS from normal ACSF (nACSF), Control (CTL) after 10 min perfusion with 4-AP in low-Mg2 ACSF, NBP after 10 min perfusion with 4-AP + NBP in low-Mg2 ACSF, and washout after 15 min washout with 4-AP in low-Mg2 ACSF, where (a) presents activity with 25 μM NBP, (b) presents activity with 50 μM NBP, (c) presents activity with 100 μM NBP, (d) presents activity with 150 μM NBP, (e) presents activity with 200 μM NBP, (f) presents activity with 300 μM NBP, and (g) presents activity with 1000 μM NBP. Note that other concentrations of NBP were recorded (data not shown).

**Figure 3:**
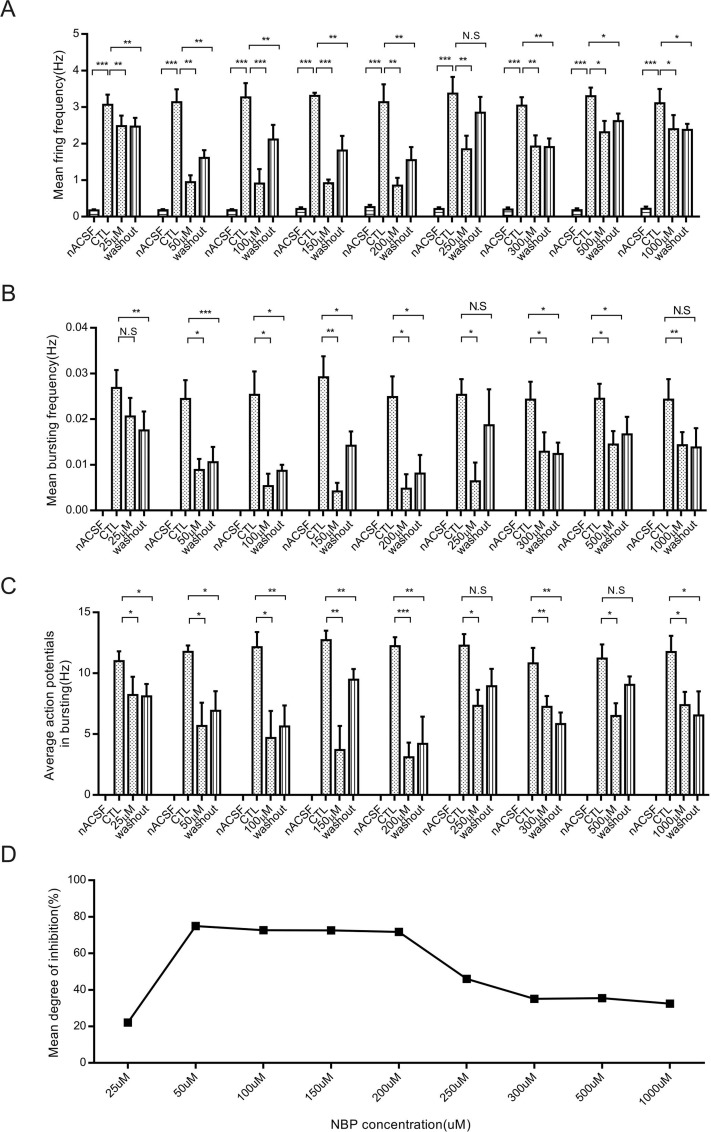
The mean firing frequency and epileptiform discharges of CA1 pyramidal neurons are reduced by NBP in 4-AP epilepsy model (**A**-**C**) Representative the bar graph of the effect of NBP on the mean firing frequency (A), mean bursting frequency (B), and average number of action potentials in bursting (C). (**D**) Representative the mean degree of inhibition in 4-AP epilepsy models. The data are presented as the means ± SEM, *n* = 6–9, ^*^ *P* < 0.05, ^**^ *P* < 0.01, ^***^ *P* < 0.001, N.S represents no statistical significance, paired Student's *t*-test.

The application of 4-AP has previously been used in epilepsy models *in vitro* to elucidate epilepsy mechanisms [19–21]. The CA1 region is one of the most vulnerable areas of epilepsy in both human and animal models and generates hyperexcitable circuits that maintain and propagate epileptic activity [22]. The paroxysmal depolarizing shift (PDS) underlying the epileptiform activity resulted from the initiation of a high-frequency burst of action potentials (Figure 2A). This burst lasted for tens of milliseconds and could be so large that it led to sodium-spike inactivation [23]. To examine whether NBP would affect the excitability of a single CA1 pyramidal neuron, we added NBP to artificial cerebrospinal fluid (ACSF) with 100 μM 4-AP to record action potentials using whole-cell patch. After hippocampal slices were perfused with NBP (50 μM) for 10 min, the mean firing frequency was significantly reduced (CTL: 3.13 ± 0.35 Hz vs. NBP: 0.94 ± 0.19 Hz, *P* = 0.008, paired Student's *t*-test, *n* = 7 cells; Figures 2Bb, 3A). In addition, to examine whether different concentrations had different or concentration-dependent effects on the excitability of pyramidal neurons, we employed other 8-concentration gradients that ranged from 25 to 100, 150, 200, 250, 300, 500 and 1000 μM NBP. The results showed that the concentrations of 100 μM (CTL: 3.30 ± 0.48 Hz vs. NBP: 1.00 ± 0.47 Hz, *P* = 0.0031, paired Student's *t*-test, *n* = 6 cells; Figure 2Bc; 3A), 150 μM (CTL: 3.31 ± 0.09 Hz vs. NBP: 0.92 ± 0.10 Hz, *P <* 0.001, paired Student's *t*-test, *n* = 9 cells; Figures 2Bd, 3A), and 200 μM (CTL: 3.13 ± 0.49 Hz vs. NBP: 0.85 ± 0.21 Hz, *P* = 0.0014, paired Student's *t*-test, *n* = 9 cells; Figures 2Be, 3A) markedly reduced the mean firing frequency and that the average inhibition level was approximately 72% (Figure 3D). However, there was no significant difference in the average inhibition of these concentrations (Figure 3D). Consistent with this result, we also identified a substantial inhibitory effect on epileptiform activity, in which the burst frequency of PDS and the average number of action potentials in the PDS were significantly reduced (Figure 3B; 3C); no significant differences were observed among the NBP concentrations of 50, 100, 150, and 200 μM. Although 250, 300, 500, and 1000 μM NBP had a certain degree of influence on excitability, these effects were independent of concentration (Figures 2B, 3A–3C). However, when we reduced the concentration of NBP to 25 μM, no significant inhibitory effect was identified (CTL: 3.064 ± 0.27 Hz vs. NBP: 2.48 ± 0.29 Hz, *P* = 0.001, paired Student's *t*-test, *n* = 6 cells; Figures 2Ba, 3A–3C). To confirm that the observed effects reflected NBP alone rather than other factors, the hippocampal slices were washed with 4-AP ACSF; this step did not achieve the restoration of firing frequency and epileptiform activity in any of the groups (Figure 3A–3C). These data suggested that NBP reduced the excitability of pyramidal neurons under epileptic conditions and that 50, 100, 150, and 200 μM NBP had substantial inhibitory effects.

### NBP significantly decreases epileptiform activity in the CA1 region

**Figure 4:**
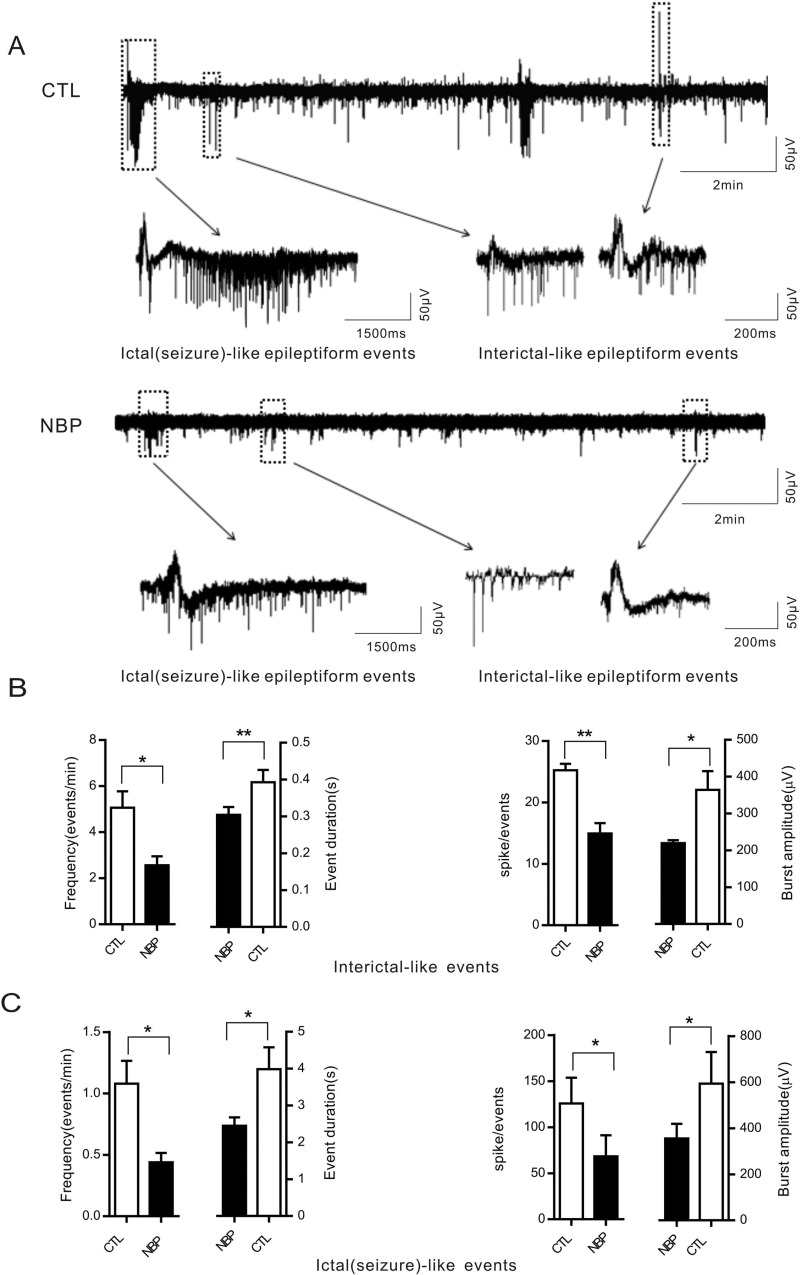
Epileptiform-like activity triggered in acute hippocampal slices significantly decreased by NBP in extracellular recordings (**A**) Representative traces of ictal-like and interictal-like epileptiform-like events triggered through the bath application of 4-AP in ACSF (CTL) and after 4-AP + 150 μM NBP. (**B**-**C**) Average values of event frequency, duration, spikes and amplitude for interictal-like events (*n* = 6 slices) (B) and ictal-like events (*n* = 5 slices) (C). The data are presented as the means ± SEM. Statistical significance (^*^ *P* < 0.05, ^**^ *P* < 0.01) was evaluated using paired Student's *t*-test (bar graph). CTL and NBP are in the same slices.

The synchronous activity of pyramidal neurons is reflected by extracellularly recorded field potentials [24]. Interictal-like epileptiform events comprise a primary population spike and subsequent secondary after-discharges, as previously described [25]. To provide additional evidence that NBP was effective at inhibiting epileptiform activity, we recorded ongoing neuronal activity in the hippocampal slice CA1 region using extracellular field potentials (Figure 4A). Because 50, 100, 150, and 200 μM concentrations of NBP had a significant inhibitory effect on neuronal activity in single pyramidal neuron and there was no significant difference in the inhibition levels of these concentrations, we selected 150 μM NBP for follow-up studies. The hippocampal slices were continuously superfused with oxygenated 4-AP ACSF for 30 min before a stable baseline was recorded for 20 min as a control. Following the addition of 150 μM NBP to the 4-AP ACSF, the data showed that significant decreases in that the following parameters of interictal-like epileptiform events: frequency (CTL: 5.07 ± 0.71 (events/min) vs. NBP: 2.57 ± 0.38 (events/min), *P* = 0.0474, paired Student's *t*-test, *n* = 6 slices; Figure 4B, left), event duration (CTL: 0.39 ± 0.03 (s) vs. NBP: 0.31 ± 0.02 (s), *P* = 0.0016, paired Student's *t*-test, *n* = 6 slices; Figure 4B, left), spike/events (CTL: 25.26 ± 1.03 vs. NBP: 14.94 ± 1.68, *P* = 0.0069, paired Student's *t*-test, *n* = 6 slices; Figure 4B, right), and burst amplitude (CTL: 364.0 ± 50.96 (μV) vs. NBP: 219.5 ± 7.74 (μV), *P* = 0.0481, paired Student's *t*-test, *n* = 6 slices; Figure 4B, right). In addition, the following ictal (seizure)-like epileptiform events showed similar effects: frequency (CTL: 1.08 ± 0.19 (events/min) vs. NBP: 0.44 ± 0.07 (events/min), *P* = 0.0401, paired Student's *t*-test, *n* = 5 slices; Figure 4C, left), event duration (CTL: 3.98 ± 0.60 (s) vs. NBP: 2.45 ± 0.23 (s), *P* = 0.0408, paired Student's *t*-test, *n* = 5 slices; Figure 4C, left), spike/events (CTL: 125.8 ± 27.90 vs. BNP: 68.40 ± 22.81, *P* = 0.048, paired Student's *t*-test, *n* = 5 slices; Figure 4C, right), and burst amplitude (CTL: 593.4 ± 137.5 μV; NBP: 355.3 ± 63.52 μV; *P* = 0.0480, paired Student's *t*-test, *n* = 5 slices; Figure 4C, right). Thus, these results indicated that NBP significantly decreased epileptiform activity in the CA1 region of hippocampal slices.

### NBP impairs spontaneous excitatory synaptic transmission in the hippocampus

**Figure 5:**
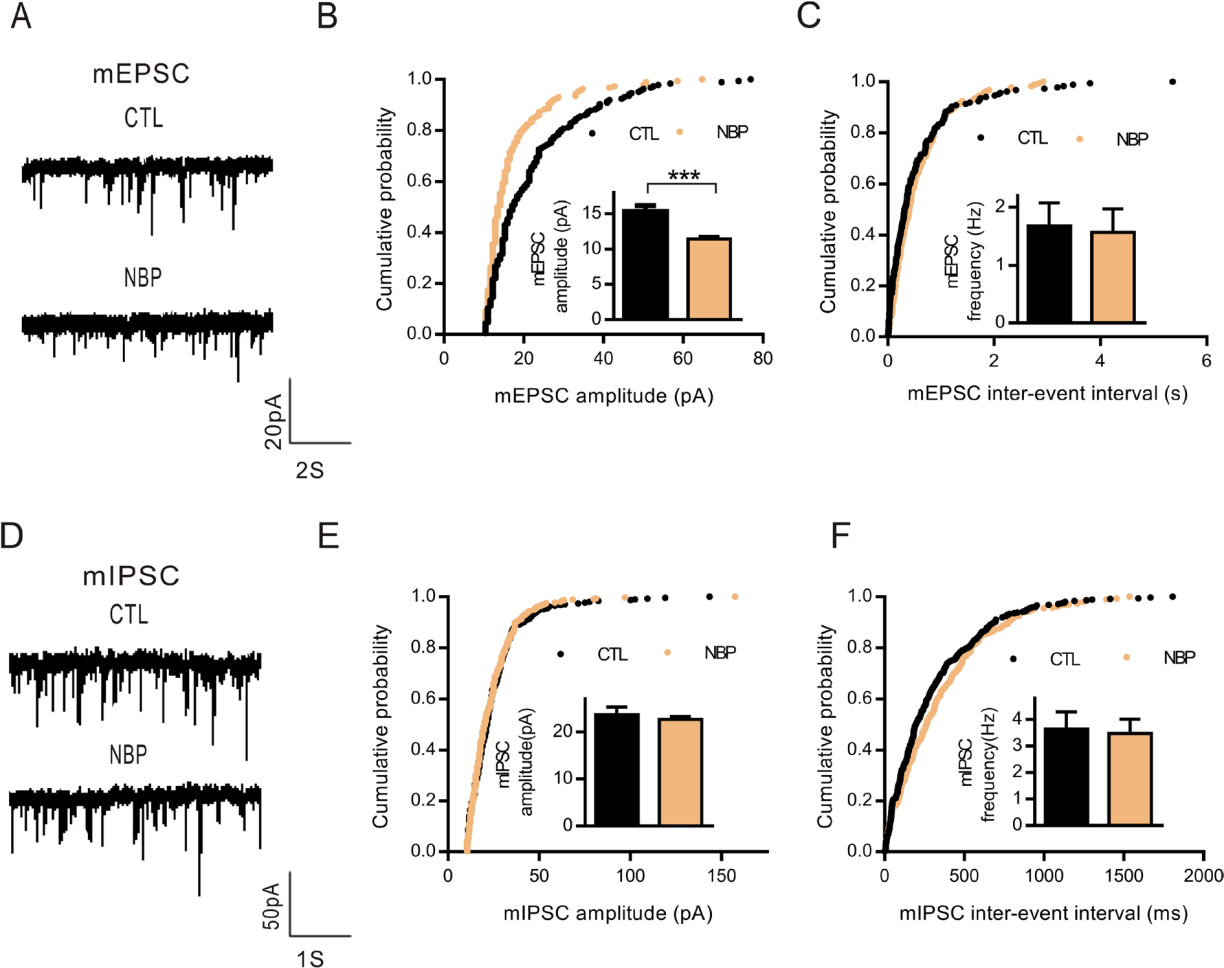
Decreased amplitudes of spontaneous mEPSCs in perfusion NBP (**A**, **D**) Representative traces of mEPSCs (A) and mIPSCs (D). (**B**, **E**) Cumulative probability curves for mEPSC (B) and mIPSC (E) amplitudes. Insets display bar graphs of the amplitudes (numbers of cells recorded; mEPSC: *n* = 9 cells; mIPSC: *n* = 11 cells). (**C**, **F**) Cumulative probability curves for mEPSC (C) and mIPSC frequency, measured based on inter-event intervals (F). Insets display a bar graph of the actual frequencies (*n* = same as for (B, E)). The data are presented as the means ± SEM. Statistical significance (^***^ *P* < 0.001) was evaluated using a KS test (cumulative probability plots) and a paired Student's *t*-test (bar graph). CTL and NBP are in the same cells.

Reduced activity in the face of cellular and group pyramidal neuron excitability suggested that the balance between total excitatory synaptic and total inhibitory synaptic events was altered by NBP. To examine whether synaptic transmission function was affected by NBP, we performed whole-cell voltage-clamp recordings at −70 mV to measure spontaneous miniature excitatory postsynaptic currents (mEPSCs) and miniature inhibitory postsynaptic currents (mIPSCs) in the CA1 pyramidal neurons of hippocampal slices (Figure 5). Notably, NBP exhibited a selective decrease in the mEPSC amplitude compared with that of the controls (CTL, 15.41 ± 0.71 pA; NBP, 11.42 ± 0.40 pA; *P* = 0.0003, paired Student's *t*-test, *n* = 9; Figure 5A; 5B) with no change in the mIPSC amplitude (CTL, 23.66 ± 1.61 pA; NBP, 22.63 ± 0.61 pA; *P* = 0.4946, paired Student's *t*-test, *n* = 11; Figure 5D; 5E). Furthermore, a cumulative probability curve indicated a leftward shift to smaller amplitudes with perfused NBP (Figure 5B; 5E), which suggested a potential impairment in the excitatory synaptic quantal size or postsynaptic receptor numbers. Moreover, no changes were identified in the mEPSC (CTL, 1.68 ± 0.40 Hz; NBP, 1.57 ± 0.41 Hz; *P* = 0.8462, paired Student's *t*-test, *n* = 9; Figure 5A; 5C) and mIPSC frequencies (CTL, 3.63 ± 0.65 Hz; NBP, 3.48 ± 0.54 Hz; *P* = 0.8036, paired Student's *t*-test, *n* = 11; Figure 5D; 5F) or in the cumulative probability curve (Figure 5C; 5F). In addition, consistent with previous reports [26–28], we confirmed that 4-AP exhibited an increased amplitude and frequency of mEPSCs and mIPSCs that resulted in a shift in the excitatory/inhibitory balance of individual CA1 pyramidal neurons to favor 4-AP actions on presynaptic terminals and postsynaptic neurons in glutamatergic and GABAergic neurons (Supplementary Figure 1). Overall, these findings suggested that NBP impaired excitatory synaptic transmission and did not alter inhibitory synaptic transmission in the hippocampus.

### NBP decreases the AMPAR/NMDAR ratio by decreasing the AMPAR-mediated component of the evoked EPSCs

**Figure 6:**
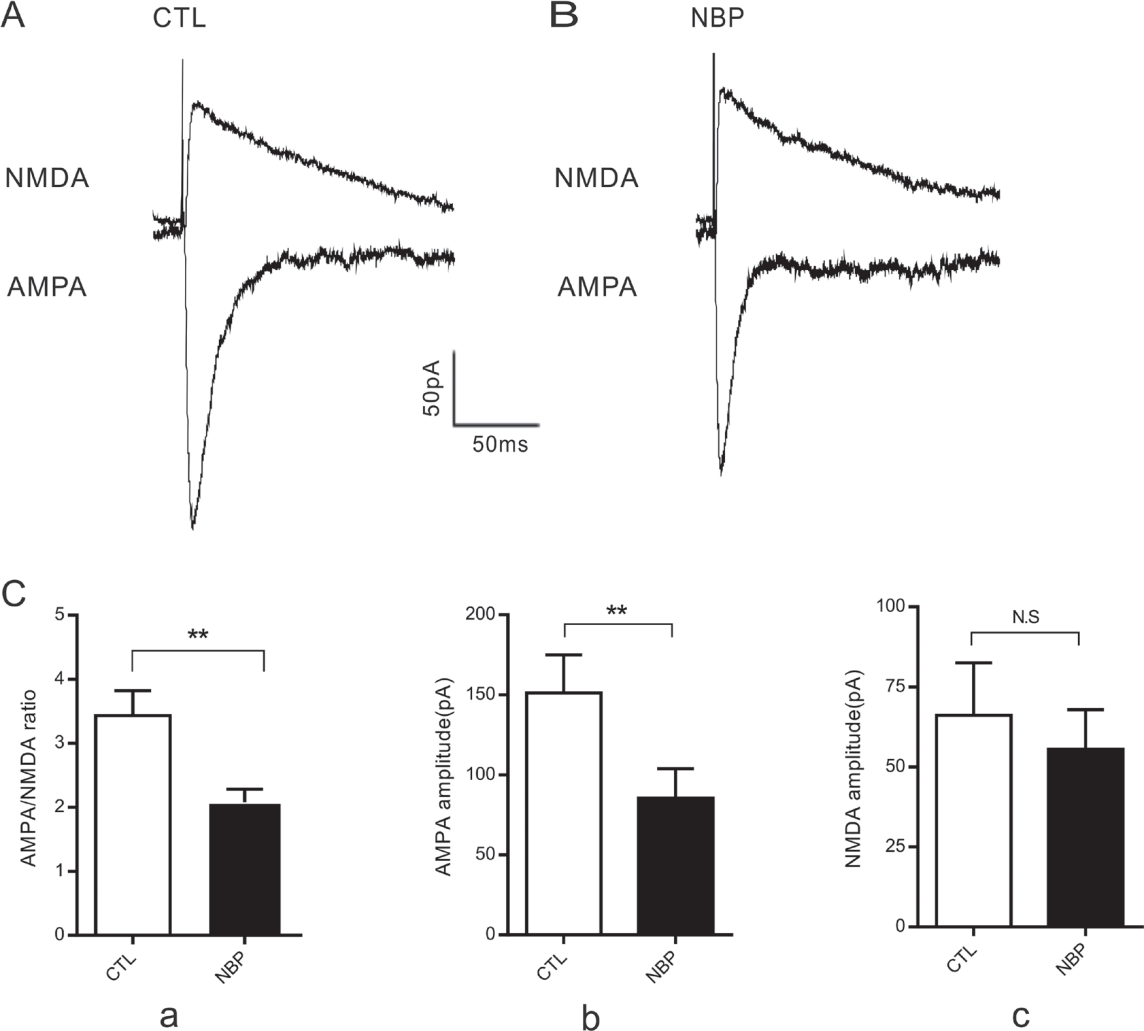
Decreased AMPA/NMDA ratio in CA1 pyramidal neurons (**A**, **B**) Representative sample traces. (**C**) Summary graph for AMPA/NMDA ratio (Ca), AMPA amplitude (Cb) and NMDA amplitude (Cc). The AMPA/NMDA ratio was determined by sequentially evaluating EPSC amplitudes at -70 mV (AMPA), followed by a + 40 mV (NMDA) holding potential in the same cell; NMDA receptor-mediated responses were measured at 50 ms post-stimulus (*n* = 6 cells). The data are presented as the means ± SEM. Statistical significance (^**^ *P* < 0.01, ^***^ *P* < 0.001) was evaluated using paired Student's *t*-test. CTL and NBP are in the same cells.

Both AMPARs and NMDARs mediate the glutamatergic postsynaptic effects at central synapses [29]. To examine whether the decreased mEPSC amplitude corresponded with a decrease in AMPAR or NMDAR numbers, we recorded AMPA and NMDA receptor-mediated synaptic currents (Figure 6). To measure the AMPAR/NMDAR ratio of excitatory postsynaptic currents (EPSCs) evoked through the stimulation of the collateral fibers of the Schaffer lateral branch, we performed whole-cell voltage-clamp recordings at a holding potential of −70 mV to measure the evoked monosynaptic AMPAR-mediated responses and at a holding potential of +40 mV to measure NMDAR-mediated responses in the same cell (Figure 6A; 6B). We determined that the AMPAR/NMDAR ratio was significantly decreased in the NBP slices compared to that of the controls (CTL, 3.43 ± 0.39; NBP, 2.08 ± 0.20; *P* = 0.0028, paired Student's *t*-test; *n* = 6; Figure 6Ca). For the latter, NBP may have decreased the AMPAR/NMDAR ratio by decreasing the AMPAR component of the synaptic response, increasing the NMDAR component of the synaptic response, or a combination of both effects. To distinguish between these possibilities, we directly assessed AMPAR-mediated and NMDAR-mediated synaptic currents. The data showed that NBP induced a significant decrease in the amplitude of AMPAR-mediated EPSCs compared to that of matched controls in the same cell (CTL, 151.2 ± 23.83 pA; NBP, 85.59 ± 18.27 pA; *P* = 0.0022, paired Student's *t*-test; *n* = 6; Figure 6Cb). Although these results suggested that the NMDAR component of the synaptic response induced by NBP was slightly reduced, the effect was not significantly different between the two groups (CTL, 66.08 ± 16.46 pA; NBP, 55.49 ± 12.40 pA; *P* = 0.0508, paired Student's *t*-test; *n* = 6; Figure 6Cc). These results indicated that the principal effect of NBP was to reduce the AMPAR component of the evoked EPSCs.

### NBP does not alter presynaptic release probability

**Figure 7:**
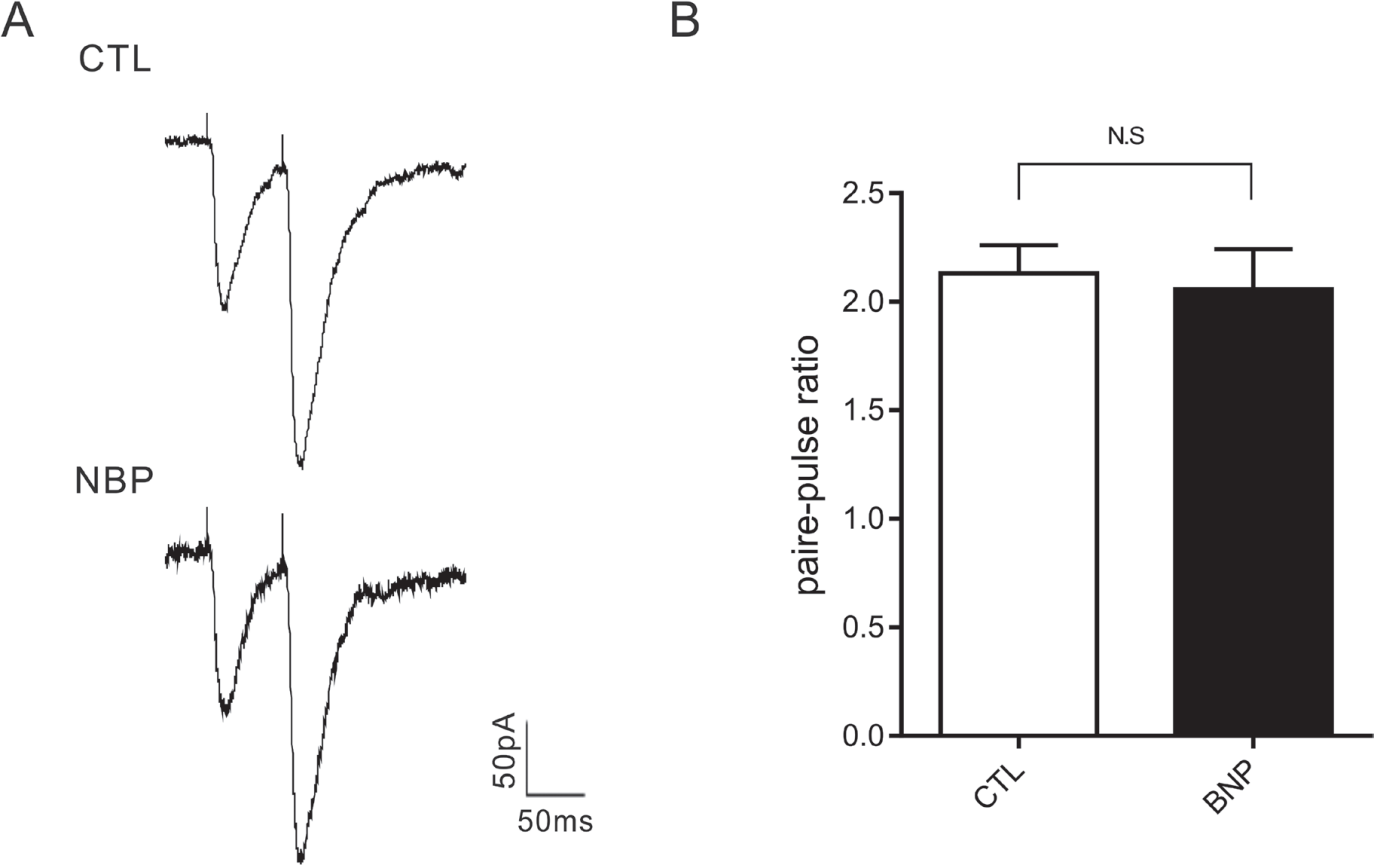
NBP did not alter the presynaptic release probability (**A**, **B**) Representative sample traces (A) and summary graphs (B) of paired-pulse facilitation measurements obtained with a 50-ms inter-stimulus (CTL vs. NBP in the same cells, *n* = 9 cells). The data are presented as the means ± SEM. N.S represents no statistical significance. Statistical significance was evaluated using a paired Student's *t*-test.

To examine whether NBP altered the probability of release at AMPAR-expressing synapses, we conducted a paired-pulse ratio (PPR) analysis (Figure 7A), a sensitive index of presynaptic release probability [30, 31]. The PPR obtained using NBP (2.06 ± 0.19; *n* = 9) did not differ from that obtained in the controls at the 50-ms intervals tested (2.13 ± 0.13; *P* = 0.6940, paired Student's *t*-test; *n* = 9; Figure 7B). These results indicated that NBP did not alter the presynaptic release probability.

### GluA2-lacking calcium-permeable AMPARs are affected by NBP

**Figure 8:**
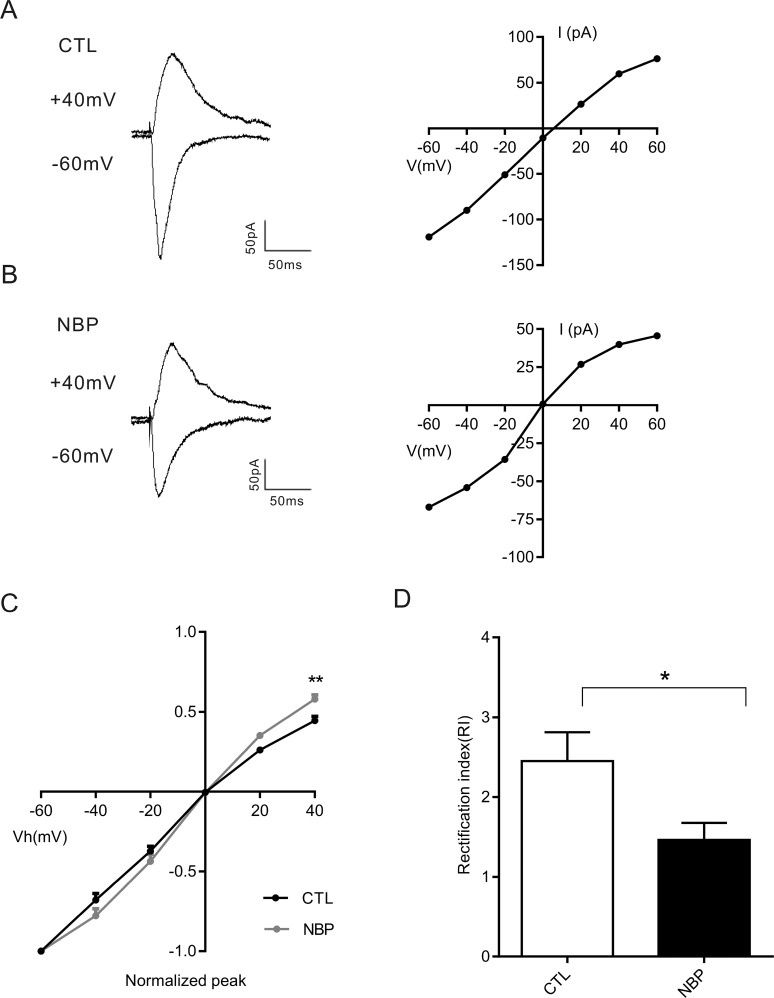
Ca2+-permeable AMPARs are involved in the NBP-mediated decrease of the AMPAR current (**A**, **B**) Representative current traces and I-V relationships of AMPA currents for control (CTL) and after NBP in CA1 pyramidal neurons at −60 and + 40 mV (Vh). (**C**) I-V curves of AMPA current amplitudes (all values for the AMPA current obtained were normalized at -60 mV as a function of holding potential (mV) show inward rectification at positive holding potentials in ordinate). (**D**) Bar plot summary of the inward rectification index (RI, the ratio of peak AMPA current amplitudes at -60 and + 40 mV) in CA1 pyramidal neurons; results indicate that the IR index is decreased after NBP treatment in pyramidal neurons (control vs. NBP in the same cells, *n* = 9 cells). The data are presented as the means ± SEM. Statistical significance (^*^ *P* < 0.05, ^**^ *P* < 0.01) was evaluated using a paired Student's *t*-test.

AMPARs mediated excitatory neurotransmission, which is critical to the generation and spread of epileptic activity and provides targets for antiepileptic drugs [32, 33]. The AMPA receptor inhibition produces powerful antiseizure activity in *in vitro* and *in vivo* models [32]. However, the functions of AMPARs are mainly depended on subunit composition. AMPARs include the major expressed subunits of GluA1, GluA2, GluA3 and GluA4, and AMPARs lacking GluA2 subunits show high Ca2+ permeability and strong inwardly rectifying (RI) in I-V relationships [34, 35]. To examine whether NBP modulated epileptiform activity through high Ca^2+^-permeability subunits, we assessed the inward rectification of CA1 region pyramidal neurons of hippocampal slices using whole-cell patch-clamp recordings. We compared the RI, measured as the ratio of mean AMPA-mediated evoked EPSCs recorded at −60 mV and +40 mV, with the control pyramidal neurons and NBP pyramidal neurons (Figure 8A; 8B). The I–V relationship of this AMPA component displayed clear inward rectification (Figure 8C). The RI of the NBP pyramidal neurons was significantly smaller (mean RI, 1.45 ± 0.21; *n* = 9) than that of the control pyramidal neurons (mean RI, 2.45 ± 0.36; *n* = 9; *P* = 0.0171, paired Student's *t*-test; Figure 8D). Thus, these data suggest that the presence of GluA2-lacking CP-AMPARs may participate in excitatory synaptic transmission to inhibit epileptiform activity.

## DISCUSSION

In the present study, we characterized the effects of NBP in an *in vivo* PTZ-induced acute epileptic seizure model and an *in vitro* 4-AP acute hippocampal slice seizure model. We found that NBP had an anticonvulsant effect and reduced neuronal hyperexcitability through postsynaptic GluA2-lacking CP-AMPARs.

Previous studies have reported that NBP can reduce the levels of excitatory neurotransmitters in epileptic mouse brain tissue [16, 17]. However, whether NBP can regulate animal behavior and the specific regulatory mechanism are unclear. Our data indicated that NBP decreased the GTCS severity and seizure-like events of LFPs in a PTZ-induced epileptic seizure model. To determine whether NBP had the same effect *in vitro*, we tested epileptiform activity in an *in vitro* 4-AP epilepsy model with acute hippocampal slices with whole-cell patch. No previous studies have shown the optimal concentration of NBP in the epileptic environment using whole-cell recording techniques; thus, we investigated the effect of different concentrations of NBP. Although reports have suggested that l-3n-butylphthalide (l-NBP) generated a maximum reduction of 70% TREK-1 currents at a concentration of 10 μM, these tests were performed in Chinese hamster ovary cells during non-epileptic conditions [36, 37]. In the present study, we determined that NBP at concentrations of 50, 100, 150, and 200 μM had a significant inhibitory effect on the spontaneous firing frequency and epileptiform activity; in general, this inhibition was not concentration dependent. At NBP concentrations greater than or equal to 250 μM, the degree of inhibition decreased, and at concentrations smaller than 50 μM, the degree of inhibition decreased. The specific mechanism is unclear, but it may be related to the number of receptors. The number of receptors is limited; when the NBP concentration increases gradually, the ratio of binding reaches saturation. However, the degree of inhibition was decreased with increasing concentrations, perhaps because of the toxic effects of NBP. The inhibition effect was partially reversed after washout at all concentrations, consistent with a previous study [36]. The potential causes may be related to the hydrophobic structure of NBP, which makes its effects difficult to completely reverse after washout. Ultimately, we used 150 μM NBP for the continuous perfusion of the hippocampal slices in the remaining experiments.

Reduced neurotransmission network excitability was accompanied by both reduced spontaneous excitatory synaptic input and increased inhibition. The recurrent nature of hippocampal circuitry greatly complicates the problem of identifying the circuit elements responsible for a change in overall circuit excitability [38]. In the present study, there were clear inhibitory effects of NBP on the mEPSC amplitude; the mIPSC frequency and amplitude were not altered, which indicates impaired excitatory synaptic transmission. Although NBP has been reported to reduce the glutamate content in the brains of epileptic mice [16], we have provided the first evidence that inhibitory synaptic activity is not altered using electrophysiological methods. Both AMPARs and NMDARs mediate the postsynaptic effects of glutamate at synapses [29]. NBP decreased the AMPA/NMDA ratio by decreasing the AMPAR-mediated component of the evoked EPSCs in the present study. Previous studies showed that NBP reduced the withdrawal symptoms of alcohol in rats through the down-regulation of NR2B mRNA expression [39] and the up-regulation of NR2B expression in streptozotocin-induced diabetic rats [40] using RT-PCR and liquid chromatography, but the experiments were performed in animals using gastric perfusion and intraperitoneal injection rather than the single systemic administration of NBP in acute hippocampal slices. This methodology is different from the conditions used in our study, and the exact mechanisms underlying these differences are currently unknown.

The PPR did not change, which suggests no alterations of the presynaptic release probability [31]. The current results indicating the absence of a change in release probability are consistent with previous studies that showed a reduction in mEPSC amplitude. Furthermore, the RI was significantly smaller in response to NBP, which suggests that GluA2-lacking CP-AMPARs participated in excitatory synaptic transmission to inhibit epileptiform activity. However, the key subunits and the idiographic action mechanism should be addressed in further studies.

In summary, NBP had an anticonvulsant effect in an *in vivo* PTZ-induced epileptic seizure model, reduced epileptiform activity in the CA1 region of hippocampal slices in an *in vitro* 4-AP epilepsy model, and selectively regulated the AMPA current through postsynaptic CP-AMPARs. NBP has been approved for stroke treatment in China and may be used in future experimental or clinical therapies in patients with epilepsy.

## MATERIALS AND METHODS

### Animals

This study was performed on Sprague Dawley male rats (150–210 g). All rats were housed in cages in a colony and maintained at 22 ± 1°C with a 12-h light/dark cycle. All experimental procedures were conducted in strict accordance with the National Institutes of Health Guide for the Care and Use of Laboratory Animals and were approved by the Ethics Committee of Chongqing Medical University (CQMU 0002648).

### Drug administration and behavioral testing in PTZ-induced acute epileptic seizure model

Experimental rats were transferred to an open field and habituated for 25 min prior to drug administration. Following the habituation period, the rats were randomly divided into two groups (*n* = 12 per group) for the intraperitoneal (i.p.) administration of vehicle (corn oil, Shanghai Aladdin Bio-Chem Technology Co., Shanghai, China) or NBP (100 mg/kg dissolved in vehicle, Chinese Academy of Life Sciences); PTZ [15] (70 mg/kg, i.p., Sigma-Aldrich, USA) was administered after 2 h. Seizures were assessed using the Racine scale [41]: stage 0, no response; stage 1, ear and facial twitching; stage 2, activation of extensors and rigidity; stage 3, myoclonic jerks with rearing; stage 4, turning over onto one side with tonic-clonic seizures; and stage 5, generalized tonic-clonic convulsions and death. The monitored parameters were recorded over a 60-min period after PTZ injection as follows: the latency to onset of the first generalized tonic–clonic seizures (time to onset of stage 5), the latency and duration of generalized tonic–clonic seizures(s), and the percentage of mortality.

### Surgical procedures and LFP recording in PTZ-induced acute epileptic rats

Surgical procedures and LFP recording were performed as previously described [42]. Under 3.5% chloral hydrate (1 ml/100 g, i.p.) anesthesia, experimental rats were secured in a stereotactic head frame (RWD Life Science Co., Ltd., Shenzhen, China). Using aseptic techniques, a midline incision was made over the cranium, and small burr holes were subsequently drilled for electrode placement. The reference electrodes were implanted in the following position: anteroposterior (AP), −3.3 mm; mediolateral (ML), –2.6 mm. The recording electrodes were implanted into the dorsal hippocampus at a depth of 2.6 mm from the surface of the skull and targeted to the stratum radiatum of CA1. The rats were allowed to recover from surgery for 7 days before LFP recording. LFPs were recorded using the OmniPlex^®^ D Neural Data Acquisition System (Plexon, Dallas, TX). The signals were filtered (0.1–1000 Hz), preamplified (1000×), and digitized at 4 kHz. The procedure for NBP administration was the same as that for behavioral testing. After recording the background activity for 30 min, we induced acute seizures using PTZ (70 mg/kg, i.p.). The LFPs were continuously recorded (more than 60 min). Seizure-like discharge events in the LFP recordings were defined as high-frequency (frequency > 5 Hz), high-amplitude (amplitude >2-fold baseline value) synchronous spike activity and/or multi-spike complexes [15, 43]. The parameters analyzed in the LFP recordings included the latency and duration of seizure-like discharge events in which the behavior showed GTCSs.

### Slice electrophysiology recordings

#### Acute hippocampal slice preparation

Hippocampal slices from Sprague Dawley rats were prepared as previously described [44, 45]. Briefly, rats were anesthetized with 3.5% chloral hydrate (100 g/ml) and decapitated. The brains were rapidly removed and placed in an ice-cold oxygenated cutting solution of ACSF that contained the following (in mM): 2.5 KCl, 1.25 NaH_2_PO_4_·2H_2_O, 10 D-glucose, 6 MgCl_2_·6H_2_O, 26 NaHCO_3_, 220 sucrose, and 0.5 CaCl_2_ at pH 7.4 (with 95% O_2_ and 5% CO_2_). After removing the frontal region of the neocortex and cerebellum, the hemispheres were separated. The brain was glued onto agar, and slices (400 μm) were transversely cut using a vibratome (VT1200S; Leica, Mannheim, Germany). The slices were incubated at 34°C for a recovery period of 60 min prior to recording.

### Whole-cell patch-clamp recording in pyramidal neurons

After recovery, all slices were placed in a recording chamber and continuously perfused at a flow rate of 1.5–2 ml/min with oxygenated ACSF that contained 100 μM 4-AP (Sigma-Aldrich, USA) added to low-Mg_2_ ACSF containing the following (in mM): 124 NaCl, 2.5 KCl, 1.25 NaH_2_PO_4_·2H_2_O, 26 NaHCO_3_, 0.5 MgCl_2_·6H_2_O, 2 CaCl_2_, and 10 glucose at pH 7.4 (with 95% O_2_, 5% CO_2_, and 300-315 mOsm/kg). Whole-cell patch-clamp recordings were performed in CA1 pyramidal neurons at room temperature (22–24°C) using a Digidata 1440A (Axon Instruments, USA) and a MultiClamp amplifier 700 B (Molecular Devices, Palo Alto, CA). Signals were digitized at 10 kHz and filtered at 2 kHz. The patch pipettes were pulled by a micropipette puller (P-97, Sutter Instrument, USA) with a resistance of 3–6 MΩ. For action potential recording, the internal solution contained (in mM): 60 K_2_SO_4_, 60 NMG, 40 HEPES, 4 MgCl_2_·6H_2_O, 0.5 BAPTA, 12 Na-phosphocreatine, 2 Na _2_-ATP, and 0.2 Na_3_-GTP. For excitatory current recording, the internal solutions contained (in mM): 130 CsMeSO_4_, 10 HEPES, 10 CsCl, 4 NaCl, 1 MgCl_2_·6H_2_O, 1 EGTA, 12 Na-phosphocreatine, 0.5 Na_3_-GTP, 5 Mg-ATP, and 5 NMG; the inhibitory-specific internal solutions contained (in mM): 100 CsCl, 10 HEPES, 1 MgCl_2_·6H_2_O, 1 EGTA, 30 NMG, 0.5 Na_3_-GTP, 5 Mg-ATP, 1 EGTA and 12 Na-phosphocreatine (pH 7.2, 280–290 mM mOsm).

In the experiments, spontaneous firing was measured under normal conditions (ACSF), epileptic conditions (low-Mg_2_ ACSF+4-AP), NBP-treated conditions (low-Mg_2_ ACSF+4-AP+NBP) and washout conditions (low-Mg_2_ ACSF+4-AP). The perfusion protocol was performed as follows: Step 1: Normal ACSF (recording 10 min) ----- basal frequency of AP; Step 2: Low-Mg_2_ ACSF+4-AP (perfusing 10 min) ----- recording 20 min; Step 3: Low-Mg_2_ ACSF+4-AP+NBP (perfusing 10 min) ----- recording 20 min; Step 4: Low-Mg_2_ ACSF+4-AP (washout 15 min) ----- recording 10 min. Epileptiform discharges were produced in 10-15 minutes by continuously perfusing 4AP with low-Mg2 ACSF; however, to reduce error and include more stable and stronger epileptic induction, we analyzed the final 10 min of recording data. The action potential was recorded at the resting membrane potential in the current clamp mode. The PDS driven by high-frequency action potentials was defined as epileptiform activity. Four or more action potentials were defined as a PDS. Three burst parameters were determined: the mean firing frequency, mean frequency of epileptiform events, and average number of action potentials in PDS [23, 46, 47].

All excitatory currents were recorded in the presence of 100 μM picrotoxin (PTX) (Sigma-Aldrich, USA), and GABAAR-mediated currents were recorded in the presence of 20 μM DNQX (Sigma-Aldrich, USA) and 50 μM AP5 (Sigma-Aldrich, USA) added to the external ACSF. In addition, the mEPSCs and mIPSCs were performed using a voltage clamp at -70 mV, and 1 μM tetrodotoxin (Shanghai Aladdin Bio-Chem Technology Co., China) was included to block action potentials during recording.

The evoked AMPAR/NMDAR peak ratio was measured in the presence of 100 μM PTX and collected at two holding potentials. The peak amplitude of evoked AMPA receptor-mediated current response was collected at a holding potential of −70 mV. Then the same neuron was voltage clamped at +40 mV, and to avoid contamination with AMPA-receptor mediated currents, the amplitude of 50 ms post-stimulus was identified as the NMDA receptor-mediated response [45]. For evoked EPSCs, a bipolar tungsten stimulation electrode (diameter: 50 μm, California Fine Wire, USA) was positioned approximately 150 μm rostral to the recording electrode to stimulate afferent fibers with the application of a stimulation pulse of 0.1 ms in duration and 0.05 Hz in frequency [48, 49].

To assess the presynaptic activity, paired-pulse facilitation experiments were performed in the presence of 100 μM PTX and 50 μM AP5. The holding potential was -70 mV. Two EPSCs were evoked through a pair of 50-ms interval stimuli. The PPR was defined as the ratio of the amplitude of the second synaptic response to the amplitude of the first synaptic response [44, 50, 51].

The rectification index of GluA2-lacking CP-AMPARs was determined using 100 μM picrotoxin and 50 μM AP5 in the ACSF and 0.1 mM spermine (Sigma-Aldrich, USA) added to the excitatory intracellular solutions [35, 44]. The slopes of the plotted I-V curves were determined by fitting straight lines over the voltage ranges of −60, –40, –20, 0, +20, +40, and +60 mV. The RI was calculated as the AMPAR-mediated current response at –60 mV/+40 mV. All values to the AMPA-R EPSC obtained were normalized at –60 mV as a function of the holding potential (mV) and show inward rectification at positive holding potentials in ordinate [44, 52].

### Extracellular recordings

Field potentials were extracellularly recorded in the pyramidal cell layers in the CA1 area of the slices. The borosilicate glass microelectrode used for extracellular recording was filled with 1 M NaCl. The recorded data were filtered at 0.1 Hz and 1 kHz and were amplified 1000X using the MultiClamp 700 B digitized at 2 kHz with Digidata 1440. To examine epileptiform activity, the hippocampal slices were continuously superfused with oxygenated 100 μM 4-AP ACSF in low-Mg_2_ ACSF at room temperature at a rate of 2–3 ml/min for 30 min before a stable baseline was recorded for 20 min as a control. After the slices were subsequently superfused with 100 μM 4-AP ACSF in low-Mg_2_ ACSF with 150 μM NBP at the same rate for 30 min, epileptiform events were recorded for an additional 20 min.

The onset of synchronous population activity began at the negative peak of the initial population spike recorded in the pyramidal cell layer [53]. The recording noise with an electrode placed in the perfusing solution was typically 4–5 μV peak-to-peak; therefore, spikes of epileptiform events with the peak-to-peak amplitude of approximately 200 μV were accepted. The duration of an epileptiform event was measured as the interval between the first and last population spike present in each epileptiform event. Events with durations < 1 s were defined as interictal-like events, and events with durations > 2 s were defined as ictal-like events, as previously reported [25, 53–55]. Ictal (seizure)-like epileptiform events typically comprised an initial sustained phase (tonic) and a subsequent phase characterized by intermittent patterns of population discharges (clonic), as previously described [25]. The Clampfit 10.3, MiniAnalysis 6.03 Synaptosoft and Origin 9.0 (Microcal Software, USA) programs were used for the acquisition and analysis of extracellular field potentials.

### Statistical analyses

Electrophysiological signals were analyzed using Clampfit 10.0.3, Mini Analysis Program, Origin 9.0, CorelDRWX4 (Corel, Canada), and GraphPad (GraphPad Software, La Jolla, California, USA). The following whole-cell recording criteria were used: cells were rejected if the Ra or Rm changed > 25% over the course of the experiment. The liquid junction potential was compensated at 10 mV. The data are expressed as the means ± SEM. The statistical significance of the data was evaluated using Student's *t*-tests. For cumulative probability plots, a Kolmogorov-Smirnov (KS) test with a significance of *P <* 0.05 was used.

## SUPPLEMENTARY MATERIALS FIGURES AND TABLES



## Abbreviations

NBP: dl-3n-butylphthalide
l-NBP: l-3n-butylphthalide
PTZ: pentylenetetrazole
GTCSs: generalized tonic-clonic seizures
4-AP: 4-aminopyridine
ACSF: artificial cerebrospinal fluid
PDS: paroxysmal depolarization shift
mEPSC: miniature excitatory postsynaptic current
mIPSC: miniature inhibitory postsynaptic current
CP-AMPARs: calcium-permeable AMPARs
PPR: paired-pulse ratio
RI: rectification index
