# Supplementary material for: Dl-3n-butylphthalide reduces epileptiform activity through GluA2-lacking calcium-permeable AMPARs in epilepsy models
Source: Oncotarget. 2017 Oct 5;8(58):98242–57. doi: 10.18632/oncotarget.21529 (PMC5716726; doi:10.18632/oncotarget.21529)
Supplement: Supplementary file 1 [file oncotarget-08-98242-s001.pdf]

Dl-3n-butylphthalide reduces epileptiform activity through GluA2-lacking calcium-permeable AMPARs in epilepsy models

SUPPLEMENTARY MATERIALS

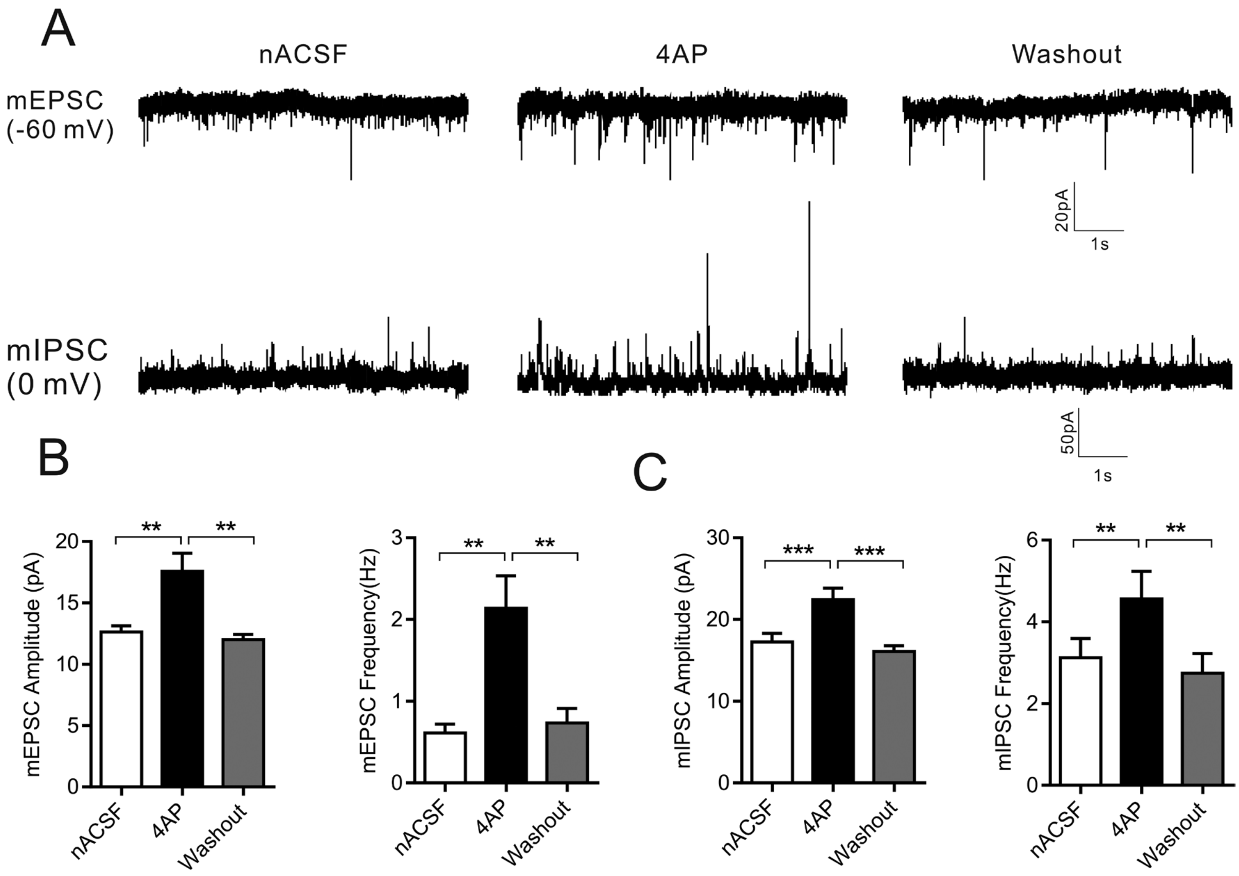

Supplementary Figure 1: Effect of 4-AP on the excitatory/inhibitory balance in the CA1 region of the hippocampus. (**A**) Representative traces of mEPSCs (-60 mV) and mIPSCs (0 mV) in the same pyramidal CA1 neuron. (**B**, **C**) Summary graphs of measurements of the amplitude (left) and frequency (right) of mEPSCs (B) and mIPSCs (C) (mEPSCs: Amplitude (pA): nACSF: 12.63 ± 0.49 vs. 4-AP: 17.56 ± 1.48, $P = 0.0047$; Washout: 12.01 ± 0.43, $P = 0.0072$; Frequency (Hz): nACSF: 0.61 ± 0.11 vs. 4-AP: 2.14 ± 0.39, $P = 0.0051$; Washout, 0.73 ± 0.18, $P = 0.004$; $n = 11$ cells. mIPSCs: Amplitude (pA): nACSF: 16.64 ± 1.09 vs. 4-AP: 23.33 ± 1.48, $P = 0.0005$; Washout: 15.91 ± 0.70, $P = 0.0002$; Frequency (Hz): nACSF: 3.21 ± 0.47 vs. 4-AP: 4.56 ± 0.67, $P = 0.0015$; Washout, 2.74 ± 0.48, $P = 0.0023$; $n = 11$ cells). The data are presented as the means ± SEM. Statistical significance was evaluated using Student's t test (* $P < 0.05$, ** $P < 0.01$, *** $P < 0.001$; N.S, no nsignificant).

MATERIALS AND METHODS

To further examine whether 4-AP specifically affects glutamatergic and GABAergic neurons, we assessed the balance of excitatory/inhibitory of individual neurons in the CA1 region of the hippocampus. Spontaneous miniature excitatory and inhibitory events in the same cell were recorded as previously described [1, 2]. The patch solution contained the following (in mM): 119 $CsMeSO_4$, 10 HEPES, 10 CsCl, 8 NaCl, 0.3 Na_3-GTP, 4 MgATP, 10 EGTA, 10 glucose, and 1 $CaCl_2$. In the same neuron, recordings were performed in normal ACSF (nACSF) by clamping at the chloride-reversal potential for GABAergic (–60 mV; to measure mEPSCs) or glutamatergic (0 mV; to measure mIPSCs); 4-AP (100 μM) was subsequently added to the normal ACSF perfused for 10 min, and the GABAergic and glutamatergic postsynaptic currents were recorded. Ultimatly, after washout by normal ACSF for 10 min, mEPSCs and mIPSCs were recorded again.

REFERENCES

1. Etherton MR, Blaiss CA, Powell CM, Südhof TC. Mouse neurexin-1alpha deletion causes correlated electrophysiological and behavioral changes consistent with cognitive impairments. Proc Natl Acad Sci USA. 2009; 106:17998–8003.
2. Dani VS, Chang Q, Maffei A, Turrigiano GG, Jaenisch R, Nelson SB. Reduced cortical activity due to a shift in the balance between excitation and inhibition in a mouse model of Rett syndrome. Proc Natl Acad Sci USA. 2005; 102:12560–65.
